# Platelet Lysate for Mesenchymal Stromal Cell Culture in the Canine and Equine Species: Analogous but Not the Same

**DOI:** 10.3390/ani12020189

**Published:** 2022-01-13

**Authors:** Alina Hagen, Heidrun Holland, Vivian-Pascal Brandt, Carla U. Doll, Thomas C. Häußler, Michaela Melzer, Julia Moellerberndt, Hendrik Lehmann, Janina Burk

**Affiliations:** 1 Equine Clinic (Surgery, Orthopedics), Justus-Liebig-University Giessen, 35390 Giessen, Germany; alina.hagen@vetmed.uni-giessen.de (A.H.); carla.u.doll@vetmed.uni-giessen.de (C.U.D.); michaela.melzer@vetmed.uni-giessen.de (M.M.); julia.moellerberndt@vetmed.uni-giessen.de (J.M.); 2 Saxon Incubator for Clinical Translation (SIKT), University of Leipzig, 04109 Leipzig, Germany; heidrun.holland@medizin.uni-leipzig.de (H.H.); vivian-pascal.brandt@uni-leipzig.de (V.-P.B.); 3 Small Animal Clinic (Surgery), Justus-Liebig-University Giessen, 35390 Giessen, Germany; thomas.haeussler@vetmed.uni-giessen.de; 4 Department of Veterinary Clinical Sciences, Small Animal Clinic, Justus-Liebig-University Giessen, 35390 Giessen, Germany; hendrik.lehmann@vetmed.uni-giessen.de

**Keywords:** platelet lysate, canine, mesenchymal stromal cells (MSC), equine, cell fitness

## Abstract

**Simple Summary:**

Regenerative medicine using platelet-based blood products or adult stem cells offers the prospect of better clinical outcomes with many diseases. In veterinary medicine, most progress has been made with the development and therapeutic use of these regenerative therapeutics in horses, but the clinical need is given in dogs as well. Our aim was to transfer previous advances in the development of horse regenerative therapeutics, specifically the use of platelet lysate for feeding stem cell cultures, to the dog. Here, we describe the scalable production of canine platelet lysate, which could be used in regenerative biological therapies. We also evaluated the canine platelet lysate for its suitability in feeding canine stem cell cultures in comparison to equine platelet lysate used for equine stem cell cultures. Platelet lysate production from canine blood was successful, but the platelet lysate did not support stem cell culture in dogs in the same beneficial way observed with the equine platelet lysate and stem cells. In conclusion, canine platelet lysate can be produced in large scales as described here, but further research is needed to improve the cultivation of canine stem cells.

**Abstract:**

Platelet lysate (PL) is an attractive platelet-based therapeutic tool and has shown promise as xeno-free replacement for fetal bovine serum (FBS) in human and equine mesenchymal stromal cell (MSC) culture. Here, we established a scalable buffy-coat-based protocol for canine PL (cPL) production (n = 12). The cPL was tested in canine adipose MSC (n = 5) culture compared to FBS. For further comparison, equine adipose MSC (n = 5) were cultured with analogous equine PL (ePL) or FBS. During canine blood processing, platelet and transforming growth factor-β1 concentrations increased (*p* < 0.05 and *p* < 0.001), while white blood cell concentrations decreased (*p* < 0.05). However, while equine MSC showed good results when cultured with 10% ePL, canine MSC cultured with 2.5% or 10% cPL changed their morphology and showed decreased metabolic activity (*p* < 0.05). Apoptosis and necrosis in canine MSC were increased with 2.5% cPL (*p* < 0.05). Surprisingly, passage 5 canine MSC showed less genetic aberrations after culture with 10% cPL than with FBS. Our data reveal that using analogous canine and equine biologicals does not entail the same results. The buffy-coat-based cPL was not adequate for canine MSC culture, but may still be useful for therapeutic applications.

## 1. Introduction

Regenerative medicine has gained tremendous attention in recent years in the veterinary field, including a growing number of studies and clinical applications in dogs. In regenerative medicine, biological materials such as cells, growth factors, and matrix substances are used to regenerate defective tissues and organs, aiming to regain their full functionality. Cell-based therapy is mostly performed with multipotent mesenchymal stromal cells (MSC), alone or combined with other biologicals. Orthobiologic blood products include autologous conditioned serum, platelet-rich-plasma (PRP), platelet concentrate, or platelet lysate (PL).

MSC-based therapies are supported by a growing body of evidence in veterinary medicine. Particularly in horses, the treatment of orthopedic diseases with MSC has a well-documented history [1,2,3,4,5,6,7]. The use of MSC in canine medicine is also becoming more popular. Canine MSC have been used successfully in several in vivo studies, especially for the treatment of osteoarthritis [8,9,10], but also for various other conditions such as atopic dermatitis [11,12], diabetes mellitus [13], inflammatory bowel disease [14], or to support neuroregeneration following vertebral compression fractures [15]. At the same time, there is a need to improve and harmonize canine MSC production processes. One of the critical factors is the in vitro cultivation of MSC before administration to the patient. In this context, the cell culture medium represents a crucial element of the cell culture process and may strongly affect efficacy and quality of therapy [16]. The critically discussed fetal bovine serum (FBS) is still the gold standard for in vitro cultivation of MSC in animal species. However, the trend in cell culture points to the use of xeno-free culture supplements, for which blood products from the same species appear most promising.

Among the orthobiologic blood products, particularly platelet products are already frequently used in clinical practice. In canine medicine, PRP or platelet concentrates have been used to treat several conditions, including osteoarthritis [17,18,19,20], lumbosacral stenosis [21], wounds [22,23], corneal ulcera [24], and aural hematoma [25]. While clinical benefits were shown, a major disadvantage of platelet concentrate is its limited long-term storage, as it cannot be frozen [26]. Furthermore, there are different methods and commercially available kits for PRP or platelet concentrate production, which limits the comparability and reproducibility of treatment results. Especially, there are major differences in terms of quality regarding the platelet and growth factor concentrations and also sterility of the product. For canine platelet therapies, it has so far been shown that the platelet concentration and leukocyte removal strongly differ between products [27,28].

PL could be a feasible off-the-shelf alternative to PRP or platelet concentrate for clinical applications [26], and at the same time represents a high-quality xeno-free replacement of FBS for cell culture [29,30]. In PL, the platelet-derived growth factors have already been released and cell membranes removed, thus PL can be stored for a long time in the freezer, while most likely offering the same benefits as platelet concentrates. Therefore, while replacing the critically discussed FBS in MSC production, positive and synergistic effects of PL might not only be achieved in cell culture, but also in the subsequent therapeutic application when combining MSC and PL. While no in vivo studies have been published using PL in dogs so far, a first study reported reduced lameness in horses that had been injected with PL for treatment of coffin joint osteoarthritis [31]. However, this benefit was transient and it is obvious that also in the case of PL, the quality of the product can vary, e.g., in terms of its growth factor concentrations, depending on the manufacturing procedures. With respect to using PL as a cell culture supplement replacing FBS, substantial progress has already been made in human medicine [30,32,33], and promising results have also been obtained in the equine species [29,34,35,36,37,38,39]. In contrast, in the canine species, studies are still rare and conflicting findings were reported when using PL for MSC culture [9,40], warranting further research.

Recently, we have established the first buffy-coat-based protocol for equine PL (ePL) production in 100% plasma devoid of additive solutions [29], which we believe improved PL production in the equine species in terms of reproducible quality and scalability. We also showed that the obtained ePL supported equine MSC expansion and basic characteristics [29]. The aim of the current study was to establish a corresponding buffy-coat-based procedure for the production of canine PL (cPL) and to evaluate its effects on canine MSC in comparison with equine MSC cultured with ePL, with the prospect of utilizing the obtained cPL in therapeutic applications as well as in MSC culture.

## 2. Materials and Methods

### 2.1. Blood Collection

Whole blood for cPL preparation was collected from 12 healthy dogs (6 males and 6 females) aged 1.4–8.1 years (median: 3.4 years; interquartile range (IQR): 3.7) after approval by the local regulatory authority (i.e., regional council Giessen, Germany, A 24/2017). The health status of the donor dogs was evaluated prior to blood collection by clinical examination and blood tests with complete blood counts from ethylenediaminetetraacetic acid (EDTA) whole blood, blood chemistry from Li-heparin blood and serum, as well as microbiological tests, as described below. For these blood tests, the blood was collected from the vena jugularis, cephalica antebrachia, or vena saphena lateralis under aseptic conditions.

After the health status was assessed, whole blood for cPL preparation was then obtained aseptically from the jugular vein. Here, 450 mL whole blood was collected from each donor in commercially available blood bags loaded with 63 mL of citrate–phosphate–dextrose (CPD; Composelect, Fresenius Kabi, Bad Homburg, Germany). In this process, the filling volume of the blood bags was standardized to 450 mL by a blood donation scale (Compoguard, Fresenius Kabi, Bad Homburg, Germany). In order to cool the temperature of the whole blood down to 20 °C within a short period of time and to improve temperature uniformity, immediately after blood collection, the blood was placed upright in a box (CompoCool^®^, Fresenius Kabi, Bad Homburg, Germany) containing butane-1,4-diol cooling plates. The blood was left there for a minimum of 2 h and a maximum of 3 h until processing in the laboratory.

### 2.2. Platelet Concentrate and Lysate Preparation

The canine whole blood was separated into its individual components of plasma, buffy coat, and erythrocyte concentrate by centrifugation in a blood separation centrifuge (Hettich Rotanta 460R, Andreas Hettich GmbH and Co.KG, Tuttlingen, Germany) at 2845× *g* for 20 min (acceleration settings 7 and deceleration settings 1) at 22 °C. Using a blood-separating device (Compomat 4G, Fresenius Kabi, Bad Homburg, Germany), the buffy coat was obtained by separating the plasma and erythrocyte concentrate using a top-bottom method. The buffy coat was left to rest for 1 h and then resuspended with 110 mL of plasma, resulting in a median buffy coat volume of 187.36 mL (IQR: 5.6) prior to the next centrifugation. The resuspended buffy coat was then centrifuged again at 266× *g* for 9.3 min, acceleration settings 7 and deceleration settings 1, at 22 °C. Subsequently, the resulting supernatant, which represented the platelet (PLT) concentrate, was recovered by the same blood-separating device. This PLT concentrate was frozen at −80 °C. Lysis of PLT to release growth factors and chemokines was induced by three freeze–thaw cycles. In this process, the PLT concentrate was thawed at 37 °C for 4 h in a dry heating device designed for thawing frozen products intended for infusion under continuous agitation (Plasmatherm, Barkey GmbH and Co., KG, Leopoldshoehe, Germany) and then refrozen at −80 °C for 20 h. Following the repeated freeze–thawing, the bags were centrifuged again in the centrifuge for blood separation at 4000× *g* for 30 min, acceleration settings 9 and deceleration settings 2, at 22 °C. To remove the cell debris, the resulting supernatant, corresponding to the lysate, was filtered through a Macopharma Plas-4 filter (Lot 11290588BM, Macopharma, Langen, Germany) using gravity. The final PLs from all dogs (n = 12) were pooled under aseptic conditions to obtain the cPL used in the cell culture experiments.

### 2.3. Microbiological Assessment

The blood samples from each animal, the PLT concentrates, and also the final pooled cPL were tested for absence of pathogens. Bacteriological analysis with Oxoid signal blood culture system (BC0100M, Oxoid Limited, Hampshire, UK) incubated at 37 °C for 7 days was performed. The cultured blood was streaked on blood agar (Blood Agar Base, Oxoid Limited, Hampshire, UK) containing 5% defibrinated sheep blood and on water-blue metachrome-yellow lactose agar (Water-blue Metachrome-yellow Lactose Agar acc. to Gassner, Sifin Diagnostics, Berlin, Germany) on days 1, 2, 4, and 6. These plates were incubated under aerobic conditions at 37 °C for 48 h. Brain–Heart Infusion Agar (Brain–Heart Infusion Agar, Oxoid Limited, Hampshire, UK) was additionally incubated under microaerobic conditions (10% CO_2_, 37 °C) for analysis after 24 and 48 h. Furthermore, we incubated Schaedler agar (BBL^TM^ Schaedler Agar, Becton Dickinson GmbH, Heidelberg, Germany) and Columbia agar (Columbia Agar (base), E. Merck, Darmstadt, Germany) for 72 h at 37 °C under anaerobic conditions in a jar using AnaeroGen™ gas bags (AnaeroGen™ 2.5 L, Oxoid Limited, Hampshire, UK). Kimmig agar (Agar for fungi (base) acc. to Kimmig modified, E. Merck, Darmstadt, Germany) was incubated for 72 h at 28 °C under aerobic conditions for selective culturing of fungi. In order to confirm the absence of mycoplasma, a 16S ribosomal RNA gene polymerase chain reaction analysis [41] was conducted.

### 2.4. Platelet and Leukocyte Counts

After the different processing steps, samples were taken to generate complete blood counts using an automated flow cytometric hematology analyzer (ADVIA 2120i, Siemens Healthcare GmbH, Erlangen, Germany) with the multispecies software MS 6.11.7. These included EDTA blood samples, citrated whole blood samples from each blood collection bag prior to further processing, PLT concentrate and lysate samples from each dog, as well as a sample from the final pooled cPL from all dogs.

### 2.5. Growth Factor Quantification and Chemical Analyses

Growth factor concentrations were analyzed in serum, PLT concentrate, and PL before and after the last filtration step from each dog, as well as in the final pooled cPL. Samples were stored at −80 °C until ELISA measurement.

The concentration of platelet-derived growth factor (PDGF-BB) was analyzed using a commercially available canine ELISA Kit (ELC-PDGFB, Ray Biotech, Norcross, GA, USA). Transforming growth factor beta 1 (TGF-β1) was quantified using a Quantikine ELISA kit (R&D Systems, Minneapolis, MN, USA). We followed the manufacturer’s instructions, which included TGF-β1 activation with hydrochloric acid for the TGF-β1 ELISA. Finally, the ELISAs were read on an Infinite M PLEX plate reader with corresponding Magellan software (Tecan Ltd., Maennedorf, Switzerland).

Chemical quality analyses, similar to those routinely performed with FBS, were also performed on the same samples. The electrolyte content was determined using a blood gas and electrolyte analyzer (Cobas b 123 POC system, Roche Diagnostics GmbH, Mannheim, Germany). The total protein and albumin contents were determined using a clinical chemistry analyzer C400 (Pentra C400 Option I.S.E, HORIBA ABX SAS, Montpellier, France).

### 2.6. MSC Culture with FBS and PL Media Supplements

To evaluate the final pooled cPL in comparison with FBS as a cell culture supplement, adipose-derived MSC were obtained from five healthy dogs aged 7 months to 8 years (median: 4 years; IQR: 4.5). The dogs used for MSC production differed from the dogs used for cPL production. For MSC recovery, subcutaneous fat was collected as a waste material from routine surgeries and collagenase digestion was used to isolate the cells in accordance with the protocol used by Gittel et al. [42]. The plastic-adherent MSC were then cultured in FBS-supplemented culture medium until cryopreservation. For cryopreservation, MSC were frozen in cryomedium consisting of Dulbecco’s modified Eagle´s medium (DMEM, 1 g/L glucose; Gibco^®^, ThermoFisher Scientific, Darmstadt, Germany) with 40% FBS and 10% dimethyl sulfoxide (DMSO, Sigma Aldrich GmbH, München, Germany) using a freezing container (Mr Frosty, Nalgene, ThermoFisher Scientific, Darmstadt, Germany) and then stored in liquid nitrogen.

The cells were thawed and seeded in DMEM supplemented with either 10% FBS (Lot: 2078409, Gibco^®^, ThermoFisher Scientific, Darmstadt, Germany) or 10 and 2.5% cPL, 1% penicillin–streptomycin, and 0.1% gentamycin. Additionally, 1 U/mL heparin–natrium (B. Braun, Melsungen, Germany) was added to the culture medium when using cPL. Until the beginning of the experiments, the MSC were maintained under standard culture conditions (humified atmosphere, 37 °C, 5% CO_2_) for one passage (P) in the respective culture medium so that possible adaptations to the medium could take place. The experiments were performed from P3 to P5.

The basic MSC characterization experiments, namely the assessment of cell proliferation and differentiation, were performed in direct comparison to corresponding equine MSC cultures. For this purpose, adipose-derived MSC from five horses aged 3 to 8 years (median: 5 years; IQR: 2) and ePL pooled from 19 other horses, produced as previously described [29], were used accordingly. Thereafter, canine MSC were further characterized with regard to apoptosis, necrosis, and senescence markers, as well as their genetic stability.

### 2.7. Cell Proliferation and Metabolic Activity

For calculation of the generation time, canine and equine MSC population doublings in P3, P4, and P5 were analyzed. For this purpose, MSC were seeded at a density of 3000 cells/cm^2^ in cell-culture-treated flasks with different culture media and incubated under standard culture conditions for 5 days. After 3 days, a medium change was performed. On day 5, MSCs were trypsinized and then counted with a hemocytometer, excluding dead cells by trypan blue staining. The following formula was used to calculate the generation time:

(1) $$\mathrm{Generation}\mathrm{time}=\frac{\mathrm{days}\mathrm{in}\mathrm{culture}}{\frac{\ln(\frac{\mathrm{cell}\mathrm{count}\mathrm{harvest}}{\mathrm{cell}\mathrm{count}\mathrm{seeding}})}{\ln2}}$$

In addition, the metabolic activity levels of canine and equine MSC were measured on days 1 and 5 of P3, P4, and P5 by performing a tetrazolium compound (MTS) assay according to the manufacturer’s instructions (CellTiter 96^®^ AQueous One Solution Cell Proliferation Assay, Promega, Mannheim, Germany). To calculate the metabolic activity, the mean absorbance on day 5 was divided by the mean absorbance on day 1.

### 2.8. Trilineage Differentiation

Prior to inducing differentiation, canine and equine MSC were cultured in 10% FBS, 10% cPL or ePL, and 2.5% cPL or ePL. In vitro differentiation of equine MSC was performed in P2 or P3, while differentiation of canine MSC was always performed in P3.

Adipogenic differentiation was induced using StemPro™ adipogenic differentiation medium (catalog number A1007001, Gibco^®^, ThermoFisher Scientific) with 0.1% gentamycin and 5% rabbit serum. The MSC were seeded at a density of 1500 cells/cm^2^ in a 24 well plate and incubated for 3 days with their standard medium, until the medium was replaced by differentiation medium. Samples were fixed after 7 days of incubation with 50% ethanol for 20 min and then stained with Oil Red O and hematoxylin counterstain. The intensity of adipogenic differentiation was determined by blinded observers using a scoring system, which included the number of differentiated cells and the size and arrangement of lipid droplets in the differentiated cells [42].

For osteogenic differentiation, MSCs were seeded at a density of 1000 cells/cm^2^ in a 24 well plate, incubated for 3 days in their standard medium, and then in osteogenic differentiation medium (catalog number A1007201, Gibco^®^, ThermoFisher Scientific) containing 0.1% gentamycin for 21 days. For analysis of extracellular mineralization, after fixation of the differentiated cells with 4% paraformaldehyde for 10 min, von Kossa staining was performed and bright-field photomicrographs were taken. On these images, the mean grayscale values were extracted using Fiji ImageJ software version 2.1.0/1.5.3c.

For chondrogenic differentiation of MSC, they were washed in PBS and chondrogenic differentiation medium (Catalog number A1007101, Gibco^®^, ThermoFisher Scientific) containing 0.1% gentamycin was added. MSC were then centrifuged at 280× *g* at 4 °C for 5 min to form a cell pellet containing 500,000 cells. The pellets were cultured in centrifuge tubes for 21 days with medium changed twice weekly and then fixed with 4% paraformaldehyde for 12 h. Paraffin sections were prepared and stained with Alcian blue and Masson’s trichrome. Bright-field photomicrographs were taken and analyzed using Fiji ImageJ software [43]. The images were color-deconvolved and binarized, then the percentages of the areas stained with each staining component were analyzed and the ratios of cartilage matrix staining and counterstaining (i.e., turquoise to violet staining for Alcian blue and bluish to red staining for Masson’s trichrome) were determined.

### 2.9. Apoptosis, Necrosis, and Senescence Assays

In canine MSC, the RealTime-Glo™ Annexin V Apoptosis and Necrosis Live-Cell Assay (Promega, Mannheim, Germany) was conducted to measure the exposure of phosphatidylserine (PS) on the outer leaflet of the cell membrane during the apoptotic process and to detect necrosis using a cell-impermeant and pro-fluorescent DNA dye. MSC were seeded at 5000 cells/cm^2^ in a 96 well plate and the assay was performed according to the manufacturer’s instructions. On day 5, the intensity of apoptosis was determined by luminescence measurement and the intensity of necrosis was determined by fluorescence measurement, using the Infinite M PLEX plate reader.

A Cellular Senescence Activity Assay (Enzo Life Sciences (ELS) AG, Lausen, Switzerland) was performed to analyze the aging process of canine MSC. MSC were seeded at a density of 3000 cells/cm^2^ in a 12 well plate and cultured for 5 days. MSC were then lysed on ice using lysis buffer containing 0.5% phenylmethylsulfonyl fluoride (PMSF) and a cell scraper. The lysate was centrifuged at 14,000× *g* for 10 min at 4 °C and then the supernatant was frozen at −80°C until all samples were collected. After thawing, SA-β-galactosidase activity was measured according to the manufacturer’s instructions using a fluorometric substrate in the Infinite M PLEX plate reader.

### 2.10. Fluorescence In Situ Hybridisation (FISH) Analyses

Cell preparation was performed on canine MSC monolayer cultures in FBS and cPL 10% medium in P5, using standard cytogenetic techniques (colcemid treatment, hypotonic treatment, and methanol–acetic acid fixation according to [44,45]), and FISH according to the manufacturer’s instructions (CSL, Sapporo, Japan) for FISH analyses on interphase cells. FISH analyses applying the *Dog Chromosome XY FISH probe* (centromeric alpha satellite DNA probe; chromosome X—spectrum red; chromosome Y—spectrum green) were accomplished in all samples cultured with FBS, but only in three canine MSC cultures after cultivation in 10% cPL, as the remaining two samples showed no visible signal patterns. In total, 827 interphase cells (54–120 per sample) were analyzed. To exclude technical artefacts in the detection of gonosome aberrations, a gonosomal chromosome loss was counted only if the other gonosome was detected.

### 2.11. Statistical Analyses

Statistical analysis was performed using IBM SPSS Statistics 28 software. Possible correlations between parameters were analyzed based on the Spearman’s rank correlation. Differences between groups were analyzed using non-parametric tests for paired samples, except for the comparisons between age groups, where the samples were not related and Mann–Whitney U tests were used. When more than two groups were compared, Bonferroni-corrected *p*-values were used for the post hoc tests. Differences were considered significant at *p* ≤ 0.05.

## 3. Results

### 3.1. Canine Platelet Lysate Production

#### 3.1.1. Absence of Pathogen Contamination

Microbial contaminations were detected neither in the canine whole blood and PLT concentrates from the different donors, nor in the final pooled cPL.

#### 3.1.2. Platelet Concentration and WBC Removal

**Figure 1:**
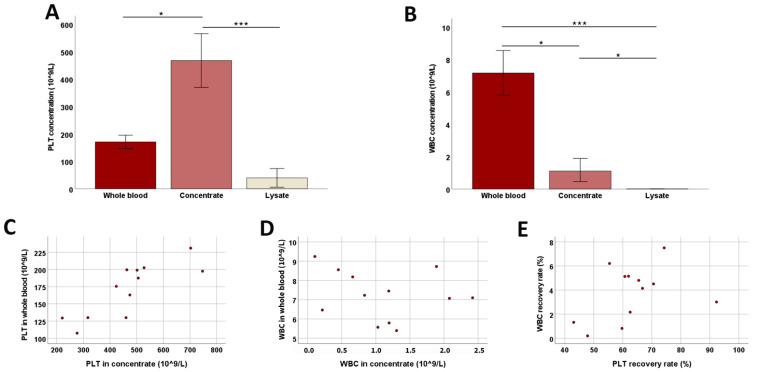
Platelet and white blood cell counts during blood processing. Median platelet (PLT; (**A**)) and white blood cell (WBC; (**B**)) counts at the different stages of blood processing (whole blood, concentrate, and lysate before filtration) are presented; error bars display the 95% confidence intervals. Friedman tests for group comparisons and subsequent post hoc tests were performed; the asterisks describe the significant differences between groups (* corresponds to *p* < 0.05; *** corresponds to *p* < 0.001). The dot plots show PLT (**C**) and WBC (**D**) counts in whole blood vs. concentrate and WBC vs. PLT recovery rates (**E**); the correlation shown in (**C**) was significant (*p* < 0.001 and r = 0.820, based on Spearman’s rank correlation). All data were obtained from n = 12 dogs.

During the preparation of cPL, a median PLT concentrate volume of 129.8 mL (IQR: 14.1) was recovered from a median whole-blood volume of 519.2 mL (IQR: 13.5), while 62.3% (IQR: 9.1) of PLTs and 4.3% (IQR: 3.1) of WBC were recovered from the whole blood. In concentrate, the PLT concentration was increased 2.6-fold compared to whole blood (*p* < 0.05) and the WBC concentration was decreased 0.2-fold (*p* < 0.05). After lysis but before filtration, the PLT number in the lysate was strongly decreased (*p* < 0.001), but still low numbers of PLT could be detected (Figure 1). The number of WBCs in the lysate was negligible compared to the concentrate (*p* < 0.05) and whole blood (*p* < 0.001). The PLT concentrations in whole blood and concentrate correlated strongly (*p* < 0.001 and r = 0.820) (Figure 1).

#### 3.1.3. Growth Factor Concentrations and Chemical Analyses

**Figure 2:**
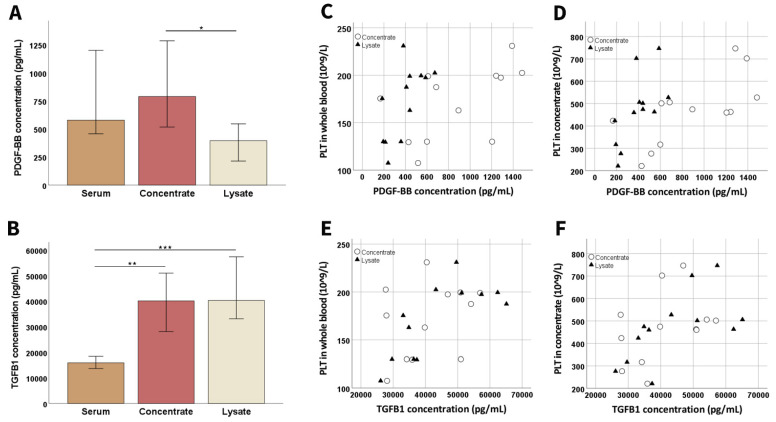
Growth factor concentrations during blood processing. Median platelet-derived growth factor (PDGF-BB; (**A**)) and transforming growth factor-β1 (TGFB1; (**B**)) concentrations at the different stages of blood processing (whole blood, concentrate, and lysate before filtration; samples had been stored at −80 °C before analysis) are presented; error bars display the 95% confidence intervals. Friedman tests for group comparisons and subsequent post hoc tests were performed; the asterisks indicate the significant differences between the corresponding groups (* corresponds to *p* < 0.05; ** corresponds to *p* < 0.01; *** corresponds to *p* < 0.001). The dot plots show platelet (PLT) concentrations in whole blood (**C**,**E**) or concentrate (**D**,**F**) vs. PDGF-BB (**C**,**D**) or TGFB1 (**E**,**F**) concentrations in concentrate (circles) and lysate (triangles); the correlations shown in (**C**) (*p* < 0.01 and r = 0.729 for concentrate; *p* < 0.05 and r = 0.641 for lysate), (**D**) (*p* < 0.01 and r = 0.804 for concentrate; *p* < 0.01 and r = 0.748 for lysate), (**E**) (*p* < 0.05 and r = 0.676 for lysate), and (**F**) (*p* < 0.05 and r = 0.671 for lysate) were significant based on Spearman’s rank correlation. All data were obtained from n = 12 dogs.

Compared to serum, the canine PLT concentrate contained higher growth factor concentrations of PDGF-BB and TGF-β1 (*p* < 0.01 for TGF-β 1). The lysate also showed significantly higher TGF-β1 concentrations compared to serum (*p* < 0.001), but the PDGF-BB concentration decreased from the concentrate to the lysate (*p* < 0.05) (Figure 2).

PLT concentration in canine whole blood showed a strong correlation with PDGF-BB in concentrate (*p* < 0.01 and r = 0.729) and a moderate correlation with PDGF-BB and TGF-β1 in lysate (*p* < 0.05 and r = 0.641 for PDGF-BB and *p* < 0.05 and r = 0.676 for TGF-β1). Similarly, PLT concentration in concentrate showed a strong correlation with PDGF-BB concentration in concentrate (*p* < 0.01 and r = 0.804) and lysate (*p* < 0.01 and r = 0.748) and also with TGF-β1 concentration in lysate (*p* < 0.05 and r = 0.671) (Figure 2).

The evaluation of the chemical composition of the samples from the different production steps showed that the pH value was not stable, as it was lower in the concentrate (*p* < 0.01) and lysate (*p* < 0.01) than in the serum. Differences were also evident in electrolyte, glucose, total protein, and albumin concentrations between serum and concentrate or lysate samples (*p* < 0.01), which is explained due to binding and/or dilution by the anticoagulant CPD, which was used in the latter (Table 1).

#### 3.1.4. Donor-Related Parameters 

**Figure 3:**
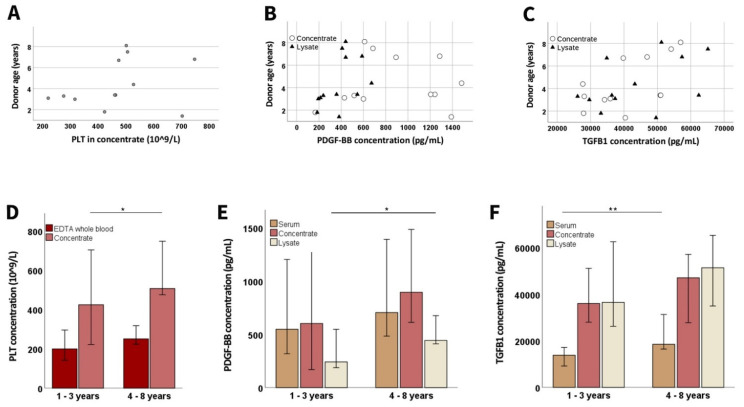
Age-related differences in platelet and growth factor concentrations. The dot plots show donor age vs. platelet (PLT; (**A**)) concentration in concentrate, as well as platelet-derived growth factor (PDGF-BB; (**B**)) and transforming growth factor (TGFB1; (**C**)) concentrations in concentrate (circles) and lysate (triangles); the correlation shown in (**B**) was significant for lysate (*p* < 0.001 and r = 0.820, based on Spearman’s rank correlation). The bar plots show the median PLT (**D**), PDGF-BB (**E**), and TGFB1 (**F**) concentrations in EDTA whole blood or serum samples drawn directly from the donor dogs, as well as in samples from different processing stages, obtained from younger and older donors; data from the younger vs. the older animals were compared using Mann–Whitney U tests; the asterisks indicate the significant differences between the corresponding groups (* corresponds to *p* < 0.05; ** corresponds to *p* < 0.01). Data were obtained from n = 7 younger and n = 5 older dogs.

There was no significant correlation between donor age and PLT concentration in whole blood or concentrate. However, the PDGF-BB concentration in lysate (*p* < 0.05 and r = 0.676) correlated positively with age, and the same trend was observed for TGF-β1. Splitting the canine donors by age groups (1–3 vs. 4–8 years) showed that the older donors had higher PLT concentrations in the concentrate (*p* < 0.05) and also higher growth factor concentrations in serum and lysate (*p* < 0.01 for TGF-β1 in serum, *p* < 0.05 for PDGF-BB in lysate) (Figure 3).

### 3.2. Growth Factor Concentrations and Chemical Compositions in the Cell Culture Supplements

The pooled cPL, the corresponding pooled ePL [29], and the FBS cell culture supplements showed some differences in their chemical analysis and composition. With possibly most relevance, there were differences in pH and in potassium, calcium, glucose, lactate, and growth factor concentrations. Regarding the latter, PDGF-BB was higher in ePL than in cPL or FBS, while TGF-β1 was much higher in cPL (Table 2).

### 3.3. Platelet Lysate in MSC Culture

#### 3.3.1. Canine and Equine MSC Morphology, Proliferation, and Metabolic Activity

**Figure 4:**
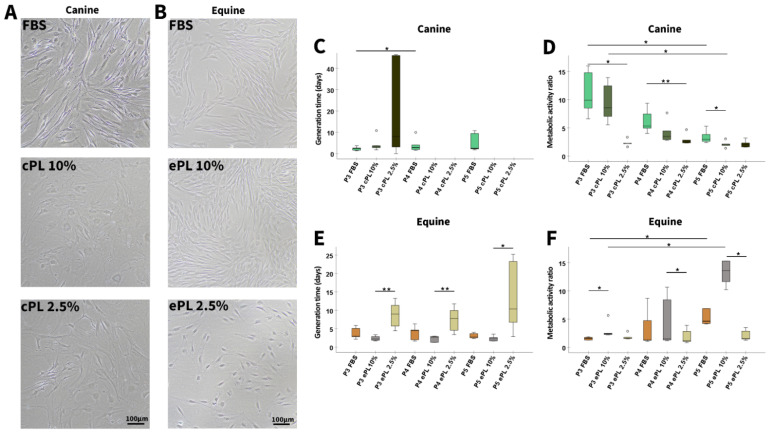
Mesenchymal stromal cell culture with different media supplements. Representative phase-contrast photomicrographs show canine (**A**) and equine (**B**) mesenchymal stromal cells (MSC) in passage 4 and at day 5 after seeding for population doubling assays in media supplemented with fetal bovine serum (FBS) or canine/equine platelet lysate (cPL/ePL). The boxplots display the generation times calculated for canine (**C**) and equine (**E**) MSC, as well as their metabolic activity as determined by MTS tetrazolium-based cell proliferation assay for canine (**D**) and equine (**F**) MSC in passages 3 to 5 (P3 to P5); note that missing generation time data were due to insufficient proliferation in these samples. Friedman tests for group comparisons and subsequent post hoc tests were performed; the asterisks indicate the significant differences between the corresponding groups (* corresponds to *p* < 0.05; ** corresponds to *p* < 0.01). Data were obtained using MSC from the same n = 5 donor dogs or horses in each group.

In FBS medium, the canine MSC showed a typical spindle shape, whereas in cPL medium, canine MSC lost the spindle shape and appeared huge, rounded, and multi-shaped. In addition, canine MSC secreted a matrix-like substance during their cultivation with 10% and 2.5% cPL medium, making detachment of MSC by trypsin difficult, as the cells formed streaks and agglomerates. In contrast to these altered characteristics of canine MSC, equine MSC showed a typical spindle shape in all three media (Figure 4A,B).

With cPL medium, due to the poor proliferation and the difficulties in MSC detachment, canine MSC counts in P4 and P5 were so low that the generation time calculation yielded negative values in several samples (3 out of 5 for cPL 10% in P4 and P5 and 3 out of 5 for cPL 2.5% in P4 and 2 out of 5 for cPL 2.5% in P5). Therefore, these data are not displayed and statistical analysis was not attempted. In the FBS group, canine MSC continued to proliferate normally, but it was evident that the generation time increased slightly over time (*p* < 0.05 from P3 to P4). In contrast, in equine MSC, the shortest generation times were observed with ePL 10% medium (*p* < 0.01 in P3 and P4 compared to ePL 2.5% in P3 and P4 and *p* < 0.05 in P5 compared to ePL 2.5% in P5), while proliferation remained consistent in FBS and ePL 10% media throughout all passages investigated (Figure 4C,E).

Confirming the findings described above, the metabolic activity of canine MSC was decreased in cPL 10% medium as compared to FBS medium (*p* < 0.05 in P5), and even more decreased in cPL 2.5% medium (*p* < 0.05 in P3, *p* < 0.01 in P4). Furthermore, the metabolic activity decreased continuously from P3 to P5 in FBS and cPL 10% media (*p* < 0.05) (Figure 4D,F).

#### 3.3.2. Canine and Equine MSC Trilineage Differentiation

**Figure 5:**
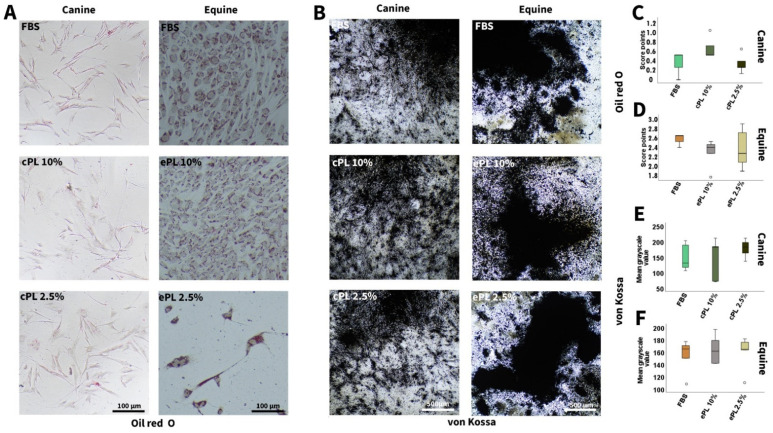
Adipogenic and osteogenic differentiation. Representative bright-field photomicrographs show canine (left column) and equine (right column) mesenchymal stromal cells (MSC) after adipogenic (**A**) and osteogenic (**B**) differentiation, with Oil Red O and von Kossa staining, respectively. Boxplots display the corresponding data obtained by scoring adipogenic differentiation of canine (**C**) and equine (**D**) MSC and image analysis using Fiji ImageJ after osteogenic differentiation of canine (**E**) and equine (**F**) MSC. MSC were cultured in the media indicated before differentiation was induced (FBS: fetal bovine serum; cPL: canine platelet lysate; ePL: equine platelet lysate). Data were obtained using MSC from the same n = 5 donor dogs or horses in each group.

**Figure 6:**
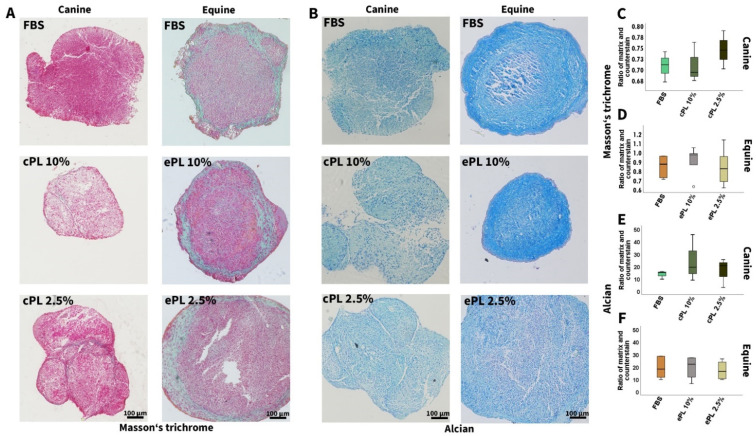
Chondrogenic differentiation. Representative bright-field photomicrographs show canine (left column) and equine (right column) mesenchymal stromal cells (MSC) after chondrogenic differentiation and Masson’s trichrome (**A**) and Alcian (**B**) staining. Boxplots display the corresponding data obtained by image analysis using Fiji ImageJ (**C**–**F**). MSC were cultured in the media indicated before differentiation was induced (FBS: fetal bovine serum; cPL: canine platelet lysate; ePL: equine platelet lysate). Data were obtained using MSC from the same n = 5 donor dogs or horses in each group.

No significant differences were observed regarding trilineage differentiation potential after MSC culture in the different media, neither in canine nor in equine MSC. However, it is of note that adipogenic and chondrogenic differentiation was weaker in canine than in equine MSC, irrespective of the medium supplement used (Figure 5 and Figure 6).

#### 3.3.3. Canine MSC Apoptosis, Necrosis, and Senescence

Despite their altered morphology, canine MSC showed less apoptosis in cPL 10% than in FBS or cPL 2.5% medium, but this was only significant when compared to the cPL 2.5% (*p* < 0.05 in P3 and P5). Additionally, significant differences over time between P3 and P5 were found, with a decrease in apoptosis with FBS but an increase with cPL 10% (*p* < 0.05).

Most necrosis was observed in cPL 2.5% MSC (*p* < 0.05 compared to FBS in P3), while no other significant differences were evident.

**Figure 7:**
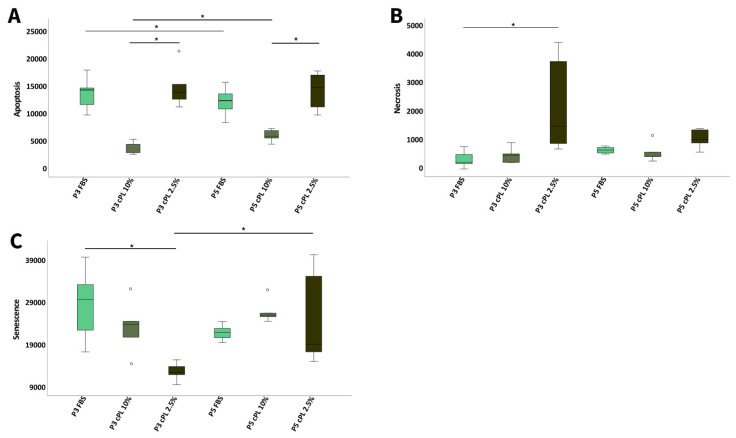
Apoptosis, necrosis, and senescence in canine cells. Boxplots display (**A**) apoptosis levels of canine mesenchymal stromal cells (MSC), measured by a luminescence-based Annexin V assay in passage 3 (P3) and 5 (P5) at day 5; (**B**) necrosis levels of canine MSC, measured using a cell-impermeant and pro-fluorescent DNA dye in passage 3 (P3) and 5 (P5) at day 5; (**C**) senescence levels of canine MSC in passage 3 (P3) and 5 (P5) at day 5, measured based on SA-β-galactosidase activity. Friedman tests for group comparisons and subsequent post hoc tests were performed; the asterisks indicate the significant differences between the corresponding groups (* corresponds to *p* < 0.05). Data were obtained using MSC from the same n = 5 donor dogs in each group.

The highest SA-β-galactosidase activity, indicating senescence, was measured in MSC cultured with FBS and then decreased via cPL 10% to cPL 2.5%, but only in P3 (*p* < 0.05 for FBS vs. cPL 2.5%). In P5, however, this trend was reversed and the senescence value was lower with FBS medium than with cPL 10% medium. The senescence values in both P3 and P5 were lowest in MSC cultured with cPL 2.5%, but with an increase between P3 and P5 (*p* < 0.05) (Figure 7).

#### 3.3.4. Canine MSC Genetic Stability

**Figure 8:**
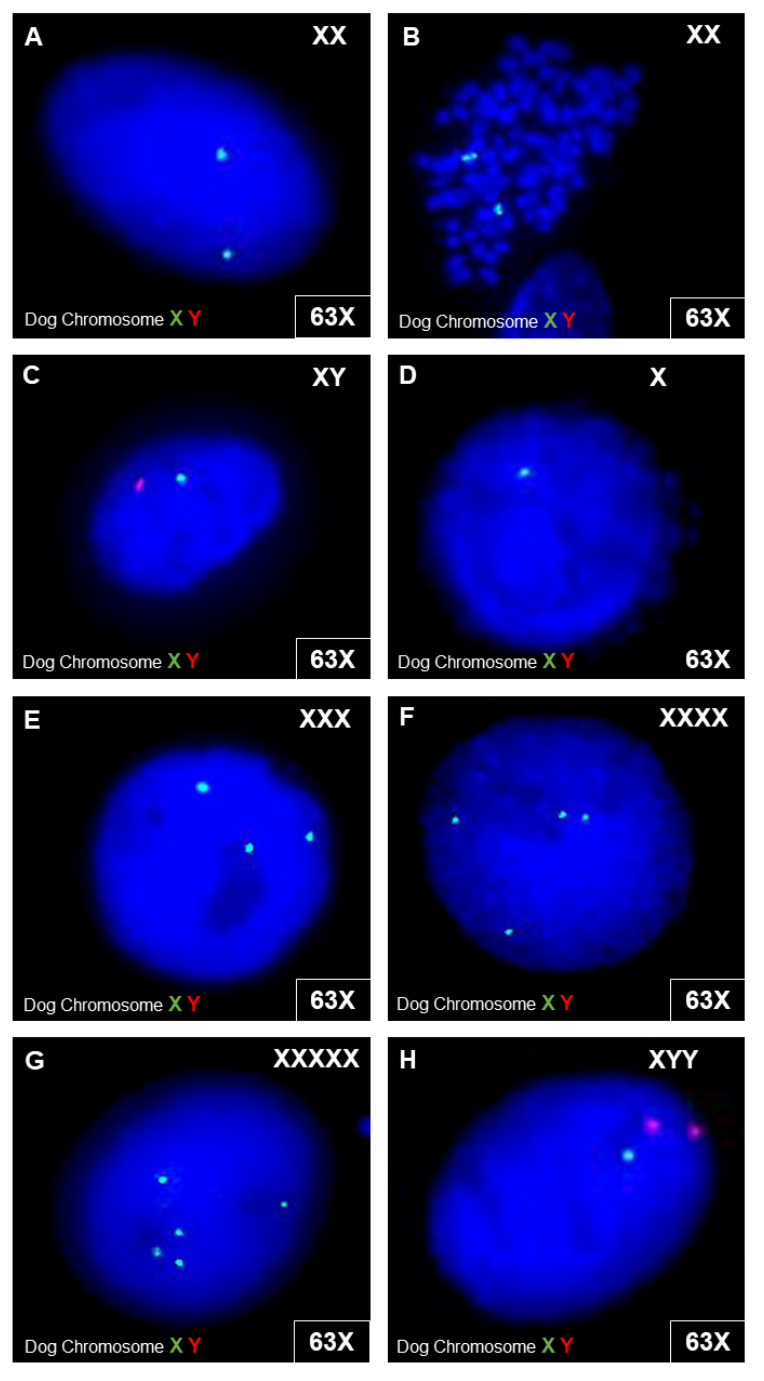
Cytogenetic analyses in canine cells. FISH analyses were performed with dog chromosome XY FISH probes (centromeric alpha satellite DNA probe; chromosome X—spectrum green; chromosome Y—spectrum red) on interphase cells (**A**,**C**–**F**) and one metaphase cell (**B**). Exemplary images show the detected signal constellations. In (**A**–**C**), microscopic images of interphase cells and metaphase cells with a normal gonosomal karyotype are shown. In (**D**–**H**), examples of interphase cells with different signal patterns are shown**,** with one signal for X (**D**), three signals for X (**E**), four signals for X (**F**), and five signals for X (**G**). For some interphase cells, a combination of signals from one X-chromosome and two Y-chromosomes were detected (**H**).

Using molecular cytogenetic analyses (FISH), canine MSC cultured with 10% cPL showed an aberrant signal pattern in 3.5–4.2% of the interphase cells analyzed. Canine MSC cultured with 10% FBS showed an aberrant signal pattern more frequently, in 6.2–10% of the interphase cells. Nevertheless, the results did not suggest the presence of clonal numeric aberrations in the gonosomes, neither in MSC cultured with cPL nor in MSC cultured with FBS. The aberrant signal patterns observed included monosomy chromosome X, trisomy chromosome X, tetrasomy chromosome X, pentasomy chromosome X, nullisomy chromosome Y, and XYY (Figure 8).

## 4. Discussion

Platelet lysate is not only an attractive off-the-shelf alternative to PLT concentrates for orthobiologic therapies, but has also shown great promise as a cell culture supplement replacing FBS in human and equine species. As data for the canine species is still scarce, in the current study, we first established a scalable procedure for cPL production from canine whole blood, building on our previously established protocol for the equine species [29]. Furthermore, we tested the effects of the obtained cPL on canine MSC in comparison to their equine counterparts, yet not with unambiguous results.

PL can be produced in three different ways regarding the PLT concentrate used as starting material. The latter can be obtained by a buffy-coat-based or a platelet-rich plasma method or directly by plateletpheresis. As the buffy coat method is best established in European human medicine [46,47], we had previously developed the first buffy-coat-based method to produce concentrate and ePL from equine whole blood collected in commercial blood bags [29]. This approach is scalable and led to similar or better results as compared to previous studies using the PRP method [34,35,36,37,39,48] or plateletpheresis [38,49]. In particular, while PLT were retained well, most WBC were removed, which we considered as an improvement compared to PRP-based protocols. Aiming to transfer this development to other relevant species, here we adapted the equine buffy-coat-based protocol [29] to generate canine PLT concentrates from whole blood. In contrast to the study presented here, the other studies on cPL which are currently available [9,40] have used PRP-based concentrates, either prepared in large volumes in blood bags [40,50] or in smaller volumes using centrifuge tubes [9]. In the presented study, we obtained a median PLT count of 469 G/L and a WBC count of 1.1 G/L in the canine concentrates. Others used concentrates with higher PLT counts for cPL production [9,40], but WBC counts, which are typically higher with the PRP as compared to the buffy coat method, were not given. There were also differences in the lysis procedures between the studies, as Russel et al. [40] used only one freeze-thaw cycle, while Lima et al. [9] used three cycles, comparable to our study. However, as no data are available on the resulting cPL in these previous studies, the outcomes cannot be compared.

Comparing our previously described ePL process [29] and the cPL manufacturing process described here and the resulting blood products, a few differences were evident. Regarding the processing, based on preliminary work with canine blood, harder centrifugation forces were used as compared to the equine protocol. Furthermore, while a hand press was used to produce the equine PLT concentrate after the second centrifugation, an automated blood separating device was used for this step in cPL production to further improve standardization [51]. The resulting concentrates had similar PLT counts in both species and even lower WBC counts in the canine concentrate as compared to the equine concentrate. Nevertheless, differences between the canine and equine blood products were observed regarding the concentrations of growth factors in the respective concentrates and lysates. This included much lower PDGF-BB concentrations but at the same time much higher TGF-β1 concentrations in canine concentrates and lysates as compared to the equine counterparts [29]. These species’ differences in growth factor concentrations and canine growth factor levels widely correspond to the literature, although the results described in different studies are diverse [52,53,54,55]. In addition, the cPL obtained after lysis showed a significantly lower PDGF-BB concentration in comparison to the canine concentrate. The TGF-β1 concentrations of the cPL were in a similar range as in the concentrate. We suggest that PLT lysis is already evident after one freezing step, which was necessary to store the concentrates until the ELISA analyses were performed, or there could be an effect of the filter or plastic surface of the storage tubes on the growth factors concentrations. This will require further studies and it could also be considered to replace the plasma in the PLT concentrate production completely or partially by additive solutions, on the one hand to preserve plasma for the patients, and on the other hand because it has already been shown that these solutions improve the stability of the product [56]. Last but not least, an interesting difference between dogs and horses was observed regarding the correlations between donor age and PLT and growth factor concentrations, which were negative in horses [29,57] but positive in dogs. This could have practical relevance with respect to the choice of donor animals for off-the-shelf PL products.

Apart from the prospect of directly using cPL in therapies, we particularly aimed to use it for MSC culture. Given that the previously used gold standard cell culture supplement, FBS, is afflicted by several problems, a reduction or replacement of FBS is necessary from both an ethical and a scientific point of view. This was also recommended by the European Medicines Agency (EMA) [58] and the International Society for Cellular Therapy (ISCT) [16]. While FBS has already been replaced by human PL in the majority of good manufacturing practice procedures in human medicine [59], and some progress has already been made in the equine field, there were only two conflicting studies on cPL in canine cell culture so far [9,40]. However, when evaluating cPL as a cell culture supplement in canine MSC culture, the results were poorer than with ePL and equine MSC, which were analyzed for comparison.

Using cPL for canine MSC culture strongly altered the cell morphology and growth behavior. In contrast, using ePL for equine MSC culture again demonstrated that when used at the same concentration (10%), ePL is a promising alternative to FBS. The canine MSC cultured with cPL, however, showed almost no resemblance with the typical spindle shape of the MSC observed when supplemented with FBS. The canine MSC cultured in cPL medium also produced a high amount of extracellular material, so that individual cells were hardly distinguishable and passaging did not lead to reliable single-cell suspensions. While the latter partly compromised the quantification of generation times, assessment of the metabolic activity confirmed that the use of cPL compromised canine MSC viability and proliferation. These disappointing results for canine MSC are in accordance with one of the two previous studies [40], although we had hoped for improvement based on the different cPL production procedures. In disagreement with our and Russell’s findings, Lima et al. [9] described canine MSC proliferation in cPL medium as improved compared to FBS medium, yet this was based on a different analytical approach with other possible influencing factors. While canine MSC expansion was only satisfactory in FBS but not in cPL medium, trilineage differentiation of canine MSC was similar after culture in FBS and cPL media. However, it should be acknowledged that canine MSC differentiation was generally rather poor, as already experienced by others [60,61,62]. Overall, considering the basic MSC characteristics, unlike equine MSC with ePL, canine MSC apparently suffered from cultivation with cPL.

To elucidate the observed effects of cPL on canine MSC culture expansion in more detail, the canine MSC were subjected to analyses of cell death, senescence, and genetic stability. Normal somatic cells, including MSC, proliferate for a limited number of doublings in culture and then reach a senescent state. In this senescent state, the cells are not dead but mitotically arrested while remaining metabolically active. Furthermore, they change their phenotype, increase significantly in size, and adopt a “fried egg morphology” [63,64,65]. If cells are too large, as observed here with cPL, they show loss of membrane and cytoskeletal integrity, increasing intracellular distances, as well as reduced surface areas for nutrient exchange and thus a reduced fitness [66]. The cell cycle of MSC can be terminated physiologically by senescence or apoptosis, which can be triggered by the same stressors [67]. The choice of pathway is strongly related to the stress level and the resulting levels of p53. Low levels of p53 promote transient cell cycle arrest and senescence. High levels of p53 and additional cooperativity of DNA-binding domains within the p53 tetramer lead to transcription of pro-apoptotic genes. In addition, at high p53 levels, pro-senescence signals are blocked, leading to apoptotic death of the cell caused by a combination of all these factors [68]. Apoptosis is the programmed cell death. In contrast, irreversible cell damage leads to passive cell death, i.e., necrosis, which in consequence leads to inflammation. In the presented study, both apoptosis and (secondary) necrosis mostly occurred in MSC cultured with 2.5% cPL. At the same time, SA-β galactosidase activity, used to indicate senescence, was lowest in these cultures. Thus, cPL used at low concentrations is likely to cause strong stress, as it initiated cell death but not senescence. However, neither increased cell death nor senescence was observed in MSC cultured with 10% cPL, and apoptosis in these cells was the lowest. This was surprising, as the canine cells showed their morphological and growth alterations not only in 2.5% but also in 10% cPL. As a final parameter for assessing cellular integrity, possible genetic aberrations in the gonosomes after cultivation with either FBS or cPL were investigated. Interestingly, canine MSC cultured with 10% cPL showed higher genetic stability than following cultivation with FBS. This is in accordance with findings in the human species [69], but was again unexpected considering the distinctly visible alterations of the MSC, which had even hampered parts of the karyotype analyses.

## 5. Conclusions

The buffy-coat-based protocol caused increased PLT concentrations and decreased WBC concentrations. Therefore, it delivers a concentrate suitable as starting material for cPL production, which could be used to offer off-the-shelf cPL therapies. However, when aiming to use the cPL obtained as MSC culture supplement, the results were not as convincing as observed with ePL and equine MSC. Even if part of the data suggested that using 10% cPL did not lead to cell damage, considering the strong alteration of MSC morphology and expansion characteristics, the use of cPL cannot be recommended for canine MSC culture in its current form.

## Figures and Tables

**Table 1:** Chemical analyses during blood processing. Data from n = 12 donors are presented as median (IQR) values.

Sample	pH	Na^+^ (mmol/L)	K^+^ (mmol/L)	Ca^2+^ (mmol/L)	Cl^-^(mmol/L)	HCO_3_ (mmol/L)	Glucose (mmol/L)	Lactate (mmol/L)	Total Protein (g/L)	Albumin (g/L)
**Serum**	7.52 (0.13)	149.15 (2.75)	4.87 (0.65)	1.33 (0.06)	110.40 (1.63)	19.60 (2.85)	3.70 (1.83)	4.95 (1.90)	64.35 (5.55)	30.10 (2.80)
**Concentrate**	7.19 (0.06)	156.80 (3.20)	2.86 (0.26)	0	81.20 (3.07)	16.65 (1.58)	26.35 (1.80)	2.45 (0.45)	47.25 (2.65)	24.35 (1.88)
**Lysate**	7.34 (0.03)	154.55 (1.50)	3.29 (0.28)	0	80.45 (2.55)	13.60 (1.85)	29.65 (0.82)	2.75 (0.53)	49.30 (3.08)	24.45 (2.33)

**Table 2:** Chemical analysis of the cell culture supplements.

Parameter	cPL ^1^	ePL ^1^	FBS
**pH**	7.33	7.52	7.45
**Na^+^ (mmol/L)**	153.70	147.80	138.60
**K^+^ (mmol/L)**	3.25	3.68	11.21
**Ca^2+^ (mmol/L)**	0	<0.10	1.268
**CL^-^ (mmol/L)**	80.70	84.50	106.60
**HCO_3_ (mmol/L)**	13.8	16.70	12.70
**Glucose (mmol/L)**	>30	23.10	2.20
**Lactate (mmol/L)**	2.70	2.90	17.70
**Total protein (g/L)**	49.20	54.10	36.80
**Albumin (g/L)**	25.50	27.90	23.00
**PDGF-BB concentration (pg/mL)**	307	3783	547
**TGF-β1 concentration (pg/mL)**	36,575	3966	3379

Note: ^1^ cPL and ePL data were obtained after the lysates from all donors (n = 12 for cPL and n = 19 for ePL) had been pooled.

## Data Availability

The data presented in this study are available on request from the corresponding author.
